# Real‐World Metabolic Improvements in Women With and Without Polyendocrine Metabolic Syndrome via a Multidisciplinary Telehealth Care Platform

**DOI:** 10.1002/edm2.70332

**Published:** 2026-10-14

**Authors:** Heather G. Huddleston, Rachel Blank, Nandita Sommers, Nicole Hellman, Felice Ramallo, Tiffany Pham, Karla Lindquist

**Affiliations:** ^1^ Department of Obstetrics, Gynecology & Reproductive Sciences University of California, San Francisco San Francisco California USA; ^2^ Allara Health New York New York USA

## Abstract

**Objective:**

To evaluate metabolic outcomes in women with and without Polyendocrine Metabolic Ovarian Syndrome (PMOS, formerly Polycystic Ovary Syndrome (PCOS))) receiving multidisciplinary care through a women's health telehealth platform that combines physician and registered dietitian support.

**Methods:**

Retrospective cohort analysis of 919 women with BMI ≥ 25 initiating care in 2024, with ≥ 6 months follow‐up (mean 9.6 months). Participants included 557 with PMOS and 362 without. Outcomes extracted from the electronic health record (EHR) included change in weight, HbA1c, HOMA‐IR, fasting insulin, fasting glucose, ALT, AST and total cholesterol between baseline and last measured value. Linear regression models adjusted for baseline values of each outcome, age, BMI, race/ethnicity and follow‐up time.

**Results:**

Significant improvements were observed across all metabolic markers for the group overall: HOMA‐IR (−14.0%), fasting insulin (−15.6%), fasting glucose (−13.0%), HbA1c (−6.2%), ALT (−21.2%), AST (−25.5%), total cholesterol (−8.0%) and weight (−4.9%). PCOS status was not associated with differential improvement in any outcome after adjustment. GLP‐1 receptor agonist use was associated with greater weight loss; however, improvements were also observed in the no‐medication group.

**Conclusions:**

Women with PCOS achieved metabolic improvements equivalent to those without PMOS through a multidisciplinary telehealth model despite presenting with a more adverse metabolic profile at baseline.

## Graphical abstract

In this retrospective cohort of 919 women with BMI ≥ 25 (557 PCOS, 362 non‐PCOS), a multidisciplinary telehealth platform produced significant improvements across eight metabolic markers over 9.6 months. Improvements were equivalent between groups despite a more adverse baseline metabolic profile in PCOS, supporting telehealth as scalable metabolic care.

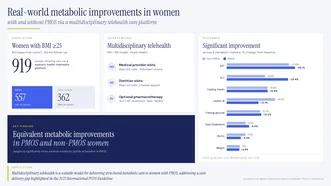

## 1 Introduction

Polyendocrine Metabolic Ovary syndrome (PMOS, formerly Polycystic Ovary Syndrome (PCOS)) affects approximately 12% of women of reproductive age and represents one of the most common endocrine disorders encountered in clinical practice. Beyond its reproductive manifestations, PMOS carries a substantial metabolic disease burden, including elevated rates of insulin resistance, hyperinsulinemia, dyslipidemia and metabolic steatotic liver disease (MASLD), and a lifetime risk of Type 2 diabetes estimated at two to three times that of the general population [1]. These heightened metabolic risks are linked to the substantial economic burden posed by this disorder [2]. Longitudinal data suggest that in the absence of structured intervention, metabolic health tends to worsen over time for women, with studies demonstrating progressive deterioration in insulin resistance, increasing rates of conversion from normoglycemia to prediabetes and rising prevalence of metabolic syndrome [3, 4]. Unfortunately, despite this progressive metabolic risk, access to the structured intervention needed to interrupt this trajectory remains limited. Women with PMOS are frequently undertreated, facing barriers including fragmented care pathways, limited access to specialists with relevant expertise and a historical tendency for medical providers to focus primarily on reproductive, but not metabolic outcomest [5].

Telehealth platforms have emerged as a promising model for delivering structured, multidisciplinary metabolic care at scale, accelerated by broad adoption following the COVID‐19 pandemic. Emerging real‐world evidence from large telehealth obesity programmes has demonstrated weight loss outcomes comparable to those seen in Phase 3 clinical trials of anti‐obesity medications, suggesting that virtual care using GLP‐1 receptor agonists can achieve meaningful clinical results outside of controlled research settings [6]. However, to date, real‐world analyses of telehealth models have largely focused on mixed‐sex adult populations treated with pharmacologic approaches. Whether similar outcomes might be achieved with a telehealth model that combines lifestyle as well as medication support has not been well‐described. Further, outcomes achieved in treating women with PMOS specifically—who may present with a more complex metabolic phenotype and different medication response profiles—have not been reported in this context.

We therefore conducted a retrospective cohort analysis of overweight and obese women with and without PMOS receiving care through a women's health telehealth platform with a focus on hormonal and metabolic health. We hypothesized that both groups would achieve improvements in metabolic health, regardless of pharmacologic treatment. We further hypothesized that despite presenting with a more adverse metabolic profile at baseline, women with PMOS would achieve improvements in key markers comparable to women without PMOS.

## 2 Methods

### 2.1 Study Design and Population

We conducted a retrospective cohort analysis of sequential new patients initiating care through a telehealth platform which focuses on hormonal and metabolic health (Allara Health) between 1 January and 31 December 2024. Eligible participants were women under 50 years of age with a BMI ≥ 25 at the time of care initiation. Follow‐up data through 1 June 2025 was considered. Only individuals with at least 6 months of follow‐up data for at least one laboratory measure were included in analyses. Participants were further required to have at least one abnormal laboratory value at baseline to be included in the respective outcome subgroup analyses, with the exception of weight, which was analysed across the full eligible cohort.

### 2.2 Telehealth Care

Allara Health is a direct‐to‐consumer telehealth platform specializing in the care of patients with complex hormonal and metabolic conditions. Patients receive care through a structured model incorporating medical provider (MD) and registered dietitian (RD) face‐to‐face visits, with most patients qualifying for insurance coverage. For the assessment of Polyendocrine Metabolic Ovary Syndrome (PMOS), Allara providers adhere to protocols that incorporate Rotterdam Criteria [1]. For patients seeking care to improve their metabolic health, baseline assessments including fasting glucose, insulin, haemoglobin a1c, lipids and liver function tests are obtained. For those with abnormal values at baseline, providers typically recommend targeted follow‐up testing, at approximately 3–6 month intervals to assess response to treatment. Weight is self‐reported and recorded at all visits. All patients have access to a smartphone app that includes recipes and educational content. Decisions regarding possible medication usage for weight and metabolic health are made jointly by the provider and patient, considering patient goals, metabolic status, side effects and cost.

### 2.3 Exposure and Predictor Variables

The primary exposure of interest was PMOS status, defined by ICD‐10 diagnosis code recorded in the electronic health record (EHR). Providers are trained to utilize the PMOS ICD‐10 codes when the patient meets Rotterdam criteria for PMOS or the patient presents with a previous physician‐based Rotterdam diagnosis. The secondary exposure of interest was pharmacologic therapy for weight and metabolic health, specifically GLP‐1 receptor agonist (GLP‐1 RA) and/or metformin. Medication status was identified through EHR‐initiated prescriptions and classified as: no medication (no GLP‐1 RA and no metformin); GLP‐1 RA only; metformin only; or both GLP‐1 RA and metformin. Covariates were identified a priori and included baseline values of each respective outcome, age, BMI, race/ethnicity and follow‐up time in months from baseline to last recorded value for each outcome.

### 2.4 Outcome Variables

Primary outcomes were change from baseline to the last recorded measurement in the following: weight (lbs), HbA1c (%), fasting insulin (μIU), fasting glucose (mg/dL), ALT (U/L), AST (U/L) and total cholesterol (mg/dL). In addition, we considered a marker of insulin resistance, homeostasis model assessment (HOMA‐IR), calculated using the standard formula: fasting insulin (μIU/mL) × fasting glucose (mg/dL)/405 [7]. All participants in this study, per inclusion criteria, had BMI ≥ 25 kg/m^2^ and were considered in the weight change analyses. Analyses for laboratory outcomes were restricted to individuals with abnormal values at baseline, defined as: HOMA‐IR > 4, HbA1c > 5.7%, fasting insulin > 24 μIU/mL, fasting glucose > 100 mg/dL, ALT > 24 U/L, AST > 24 U/L and total cholesterol > 200 mg/dL.

### 2.5 Statistical Analyses

Baseline characteristics were summarized using means and standard deviations for continuous variables and frequencies with proportions for categorical variables. Differences between PMOS and non‐PMOS groups were assessed using two‐sided *t*‐tests for continuous variables and chi‐squared tests for categorical variables. Within‐group percent change from baseline was assessed using two‐sided *t*‐tests separately in the PMOS and non‐PMOS groups. A Bonferroni correction was applied to account for the two comparisons per outcome.

Linear regression models were used to estimate the association between change in each outcome and the primary exposure (PMOS status) and secondary exposure (medication status). Models were adjusted for main effects of PMOS status and medication group, baseline value of the respective outcome, baseline age, baseline BMI, race/ethnicity and follow‐up time. Interactions between PMOS status and medication group were tested for each outcome using likelihood ratio tests. Interaction *p* values were adjusted for the eight interaction tests using the Bonferroni method. Adjusted *R* ^2^ values and predicted mean changes with 95% confidence intervals were estimated from main effects models. For the weight outcome model, baseline weight was substituted for baseline BMI given their collinearity. Predicted mean changes with 95% confidence intervals by PMOS and medication group were estimated from models incorporating interaction terms and all other covariates. All analyses were conducted in R (version 4.3.2).

### 2.6 Ethical Approvals

This study was reviewed by the Biomedical Research Alliance of New York (BRANY) Institutional Review Board (protocol #24‐330‐1249) and granted exempt status as it involved retrospective analysis of de‐identified patient data.

## 3 Results

### 3.1 Study Population and Baseline Characteristics

A total of 3117 women initiated care between 1 January and 31 December 2024. All patients had opportunity for 6 months of follow‐up. After excluding patients (*n* = 2197) without at least 6 months of follow‐up data for at least one laboratory value (calculated as time between first measurement and last measurement) the analytic cohort included 919 women. Baseline characteristics of the included analytic cohort are shown in Table 1, and a comparison of included and non‐included women is shown in Table S1. Included patients were more likely to have PMOS and had a more adverse metabolic profile. Of those included, mean age was 34.6 (SD 5.6) years, mean BMI was 34.6 (SD 7.2) and mean follow‐up was 9.6 (SD 2.6) months. More than half of the cohort (60.6%) carried a diagnosis of PMOS. Women with PCOS were younger (33.5 vs. 36.3 years, *p* < 0.001), had slightly higher BMI (35.4 vs. 33.4, *p* < 0.001), and were more likely to be prescribed metformin or combination therapy with metformin and GLP‐1RA (*p* < 0.001). The proportion receiving GLP‐1 RA alone was similar between groups (8.8% PMOS vs. 15.2% non‐PMOS). Visit frequency and follow‐up duration were comparable across groups (MD visits 6.6 vs. 6.7, *p* = 0.500; RD visits 5.8 vs. 5.7, *p* = 0.671). At baseline, women with PMOS had significantly higher rates of elevated HOMA‐IR (50.6% vs. 32.9%, *p* < 0.001), fasting insulin (29.3% vs. 15.2%, *p* < 0.001), ALT (36.3% vs. 18.8%, *p* < 0.001), AST (18.1% vs. 10.8%, *p* = 0.002), low HDL (41.5% vs. 30.7%, *p* = 0.001) and elevated triglycerides (28.0% vs. 21.8%, *p* = 0.04). Rates of elevated HbA1c, fasting glucose and total cholesterol were similar between groups.

**TABLE 1:** Baseline characteristics and outcome subgroup frequencies.

Characteristic	All (*N* = 919)	Non‐PMOS (*N* = 362)	PMOS (*N* = 557)	*p* *
Medications, *n* (%)				< 0.001
No GLP‐1 or Metformin	112 (12.2)	69 (19.1)	43 (7.7)	
GLP‐1 only	104 (11.3)	55 (15.2)	49 (8.8)	
Metformin only	381 (41.5)	132 (36.5)	249 (44.7)	
Both GLP‐1 and Metformin	322 (35.0)	106 (29.3)	216 (38.8)	
Age (years), mean (SD)	34.6 (5.6)	36.3 (5.6)	33.5 (5.3)	< 0.001
Number of MD visits, mean (SD)	6.6 (2.3)	6.7 (2.3)	6.6 (2.2)	0.500
Number of RD visits, mean (SD)	5.8 (3.6)	5.7 (3.7)	5.8 (3.6)	0.671
Follow‐up time, mean (SD)	9.6 (2.6)	9.6 (2.6)	9.6 (2.6)	0.837
Race, *n* (%)				0.038
Asian	50 (5.4)	17 (4.7)	33 (5.9)	
Black	49 (5.3)	25 (6.9)	24 (4.3)	
Hispanic	110 (12.0)	46 (12.7)	64 (11.5)	
White	393 (42.8)	140 (38.7)	253 (45.4)	
Other/multiethnic	41 (4.5)	11 (3.0)	30 (5.4)	
Unknown	276 (30.0)	123 (34.0)	153 (27.5)	
BMI, mean (SD)	34.6 (7.2)	33.4 (6.9)	35.4 (7.3)	< 0.001
Weight, mean (SD)	205.3 (45.1)	198.6 (43.2)	210.0 (45.8)	< 0.001
Outcome subgroup				
HOMA‐IR > 4, *n* (%)	401 (43.6)	119 (32.9)	282 (50.6)	< 0.001
HbA1c > 5.7, *n* (%)	202 (22.0)	83 (22.9)	119 (21.4)	0.576
Fasting insulin > 24, *n* (%)	218 (23.7)	55 (15.2)	163 (29.3)	< 0.001
Fasting glucose > 100, *n* (%)	165 (18.0)	74 (20.4)	91 (16.3)	0.113
ALT > 24, *n* (%)	270 (29.4)	68 (18.8)	202 (36.3)	< 0.001
AST > 24, *n* (%)	140 (15.2)	39 (10.8)	101 (18.1)	0.002
Total cholesterol > 200, *n* (%)	403 (43.9)	159 (43.9)	244 (43.8)	0.972
HDL < 50, *n* (%)	342 (37.2)	111 (30.7)	231 (41.5)	0.001
LDL > 130, *n* (%)	285 (31.0)	108 (29.8)	177 (31.8)	0.534
Triglycerides > 150, *n* (%)	235 (25.6)	79 (21.8)	156 (28.0)	0.036

* *p* value by *t*‐test for continuous variables and *c* ^2^ for categorical variables.

### 3.2 Metabolic Outcomes for Women With and Without PCOS

Unadjusted percent change in metabolic health outcomes is shown in Table 2. Statistically significant improvements from baseline were observed across all metabolic outcomes, overall and within both groups, following Bonferroni correction. Overall, the cohort achieved mean reductions of 14.0% in HOMA‐IR, 15.6% in fasting insulin, 13.0% in fasting glucose, 6.2% in HbA1c, 21.2% in ALT, 25.5% in AST, 8.0% in total cholesterol and 4.9% in weight.

**TABLE 2:** Baseline and change in outcome values over time for subgroups with abnormal values at baseline.

Outcome	Threshold	Overall		Non‐PMOS		PMOS		*p* value for PCOS versus non‐PMOS change values*
		Baseline	% Change	Baseline	% Change	Baseline	% Change	
Weight (lbs), mean (SD)	≥ 25	205.3 (45.1)	−4.9 (7.3)	198.6 (43.2)	−5.0 (7.5)	210.0 (45.8)	−4.8 (7.1)	0.757
HOMA‐IR, mean (SD)	> 4	7.4 (5.2)	−14.0 (63.1)	7.2 (5.7)	−18.5 (42.3)	7.5 (4.9)	−12.1 (70.0)	0.259
HbA1c, mean (SD)	> 5.7	6.1 (0.6)	−6.2 (9.8)	6.2 (0.8)	−7.2 (9.4)	6.1 (0.4)	−5.4 (10.0)	0.195
Fasting insulin, mean (SD)	> 24	38.6 (19.5)	−15.6 (49.0)	20.7 (37.2)	−15.6 (36.9)	39.0 (19.2)	−15.6 (49.0)	0.992
Fasting glucose, mean (SD)	> 100	112.2 (21.5)	−13.0 (13.2)	114.0 (28.0)	−12.7 (14.1)	110.6 (14.2)	−13.2 (12.4)	0.804
ALT, mean (SD)	> 24	39.6 (19.9)	−21.2 (40.9)	39.8 (25.4)	−14.6 (50.6)	39.6 (17.7)	−23.4 (36.9)	0.188
AST, mean (SD)	> 24	34.4 (14.7)	−25.5 (29.8)	33.7 (16.1)	−23.8 (28.1)	34.7 (14.2)	−26.1 (30.5)	0.669
Total cholesterol, mean (SD)	> 200	230.1 (33.5)	−8.0 (14.1)	230.8 (26.6)	−7.2 (13.4)	229.6 (37.3)	−8.5 (14.5)	0.373

*Note:* All change values, overall and for PCOS and non‐PCOS, were significantly different from zero based on two‐sided *t*‐tests, with Bonferroni correction applied to the non‐PCOS and PCOS *p* values.

* *p* values comparing non‐PCOS vs. PCOS % change values by two‐sided *t*‐tests.

Unadjusted comparisons of percent change between PMOS and non‐PMOS groups showed no significant differences across outcomes (all *p* > 0.05, Table 2).

### 3.3 Association Between Change in Outcome Values, Exposures and Covariates

Table 3 shows regression coefficients for exposures, covariates and outcome values. Values were adjusted for PMOS and medication exposure, baseline value of the respective outcome, age, race, BMI and follow‐up time. In adjusted analyses, higher baseline value was associated with greater change for all outcomes and higher BMI at baseline was associated with less change in HOMA‐IR and fasting insulin. Black race was associated with greater reductions in total cholesterol. Compared to no pharmacologic weight loss support, GLP‐1 RA use was significantly associated with greater weight loss in adjusted models, whether prescribed alone (−10.1 lbs, 95% CI −14.0, −6.2) or in combination with metformin (−7.1 lbs, 95% CI −10.4, −3.9). Metformin alone was not associated with significant weight change compared to no medication. All medication groups (GLP‐1 only, Metformin only or both) were also associated with greater reductions in HbA1c compared to no medication. Nevertheless, medication group was not significantly associated with a change in HOMA‐IR, fasting insulin, fasting glucose, liver enzymes or total cholesterol after adjustment. Increasing age was associated with increasing HbA1c (0.02%, 95% CI 0.01, 0.03), while higher BMI at baseline was associated with increases in HOMA‐IR (0.11, 95% CI 0.05, 0.16) and fasting insulin (0.54 mU/mL, 95% CI 0.27, 0.82). Overall, adjusted *R* ^2^ values ranged from 0.20 for weight to 0.69 for fasting glucose, reflecting greater model‐explained variance for laboratory outcomes relative to weight change.

**TABLE 3:** Regression coefficients (95% CI) for the association between change in outcome values, exposures and covariates.

Characteristic, *b* (95% CI)	Weight (*N* = 870)	HOMA‐IR > 4 (*N* = 401)	HbA1c > 5.7 (*N* = 202)	Insulin > 24 (*N* = 218)	Glucose > 100 (*N* = 165)	ALT > 24 (*N* = 270)	AST > 24 (*N* = 140)	Tot chol > 200 (*N* = 403)
PCOS vs. No PCOS	0.09 (−1.92, 2.10)	0.47 (−0.40, 1.34)	0.08 (−0.06, 0.22)	0.22 (−5.01, 5.46)	−0.88 (−5.03, 3.28)	−1.73 (−6.61, 3.15)	1.24 (−2.82, 5.30)	−5.12 (−12.12, 1.89)
GLP‐1 only vs. no GLP‐1/Met	−10.12 (−13.99, −6.24)*	−0.20 (−1.99, 1.60)	−0.57 (−0.86, −0.28)*	0.42 (−9.78, 10.61)	−7.15 (−16.24, 1.94)	−0.96 (−9.72, 7.80)	−1.92 (−9.28, 5.43)	−8.55 (−21.31, 4.20)
Met only vs. no GLP‐1/Met	0.05 (−3.07, 3.17)	−0.11 (−1.61, 1.39)	−0.27 (−0.50, −0.05)	0.66 (−7.86, 9.17)	−1.96 (−8.79, 4.86)	2.91 (−4.27, 10.08)	0.76 (−4.97, 6.49)	1.81 (−8.55, 12.17)
GLP‐1 + Met vs. no GLP‐1/Met	−7.14 (−10.41, −3.87)*	0.01 (−1.49, 1.51)	−0.47 (−0.69, −0.24)*	0.50 (−8.13, 9.13)	−4.85 (−11.56, 1.85)	2.81 (−4.41, 10.02)	1.09 (−4.73, 6.91)	−8.18 (−18.92, 2.55)
Baseline value	−0.06 (−0.08, −0.04)*	−0.76 (−0.84, −0.68)*	−0.82 (−0.93, −0.70)*	−0.83 (−0.94, −0.71)*	−0.84 (−0.94, −0.74)*	−0.61 (−0.72, −0.51)*	−0.84 (−0.96, −0.72)*	−0.78 (−0.87, −0.68)*
Baseline age	−0.02 (−0.19, 0.15)	−0.05 (−0.12, 0.02)	**0.02 (0.01, 0.03)**	−0.29 (−0.70, 0.12)	0.19 (−0.16, 0.54)	0.16 (−0.19, 0.52)	0.32 (−0.01, 0.65)	0.22 (−0.39, 0.83)
Baseline BMI	(see note)	0.11 (0.05, 0.16)*	0.00 (−0.01, 0.01)	0.54 (0.27, 0.82)*	0.07 (−0.19, 0.33)	0.26 (−0.03, 0.55)	0.13 (−0.13, 0.38)	0.10 (−0.43, 0.62)
Follow‐up months	−1.03 (−1.31,‐0.75)* −	0.04 (−0.10, 0.18)	−0.02 (−0.05, 0.00)	−0.35 (−1.13, 0.43)	0.05 (−0.64, 0.74)	0.10 (−0.50, 0.71)	−0.31 (−0.85, 0.23)	−0.07 (−1.13, 0.98)
Asian vs. White	1.90 (−2.33, 6.13)	0.87 (−0.84, 2.59)	0.08 (−0.17, 0.33)	3.53 (−5.52, 12.58)	−1.28 (−10.08, 7.52)	−0.94 (−9.73, 7.85)	−2.81 (−10.99, 5.37)	4.79 (−8.80, 18.39)
Black vs. White	−0.03 (−4.43, 4.36)	1.07 (−0.64, 2.77)	−0.09 (−0.32, 0.14)	−1.02 (−9.69, 7.64)	−11.78 (−25.27, 1.70)	−3.16 (−15.17, 8.84)	0.46 (−11.50, 12.41)	−14.41 (−28.69, −0.14)
Other vs. White	1.52 (−0.45, 3.49)	0.37 (−0.46, 1.19)	0.00 (−0.16, 0.16)	0.36 (−4.40, 5.12)	1.63 (−2.57, 5.83)	2.20 (−2.05, 6.45)	2.70 (−0.92, 6.32)	3.78 (−2.84, 10.40)
PMOS × Medication *p* value	1.000	1.000	0.090	1.000	1.000	1.000	1.000	1.000
Adjusted *R* ^2^	0.196	0.490	0.590	0.503	0.690	0.350	0.591	0.401
Adjusted mean change (95% CI)	−10.48 (−11.43, −9.52)	−1.66 (−2.05–1.28)	−0.41 (−0.48, −0.35)	−8.51 (−10.64, −6.37)	−16.18 (−18.12, −14.24)	−10.36 (−12.34, −8.38)	−10.59 (−12.28, −8.91)	−20.3 (−23.38, −17.22)

*Note:* Interaction *p* values were adjusted for the eight interaction tests using the Bonferroni method. * indicates where the corrected *p* value is < 0.05. Since BMI and weight are strongly correlated, only baseline weight was included in the weight outcome model. Race/ethnicity group ‘Other’ includes Hispanic, Native American, Other and Unknown responses.

### 3.4 Outcomes by PMOS and Medication Group

Outcomes stratified by PMOS and medication group are shown in Table S2 and Figure 1. An interaction between PMOS status and medication group was observed for HbA1c. Although the interaction was statistically significant for HbA1c in the primary analysis (likelihood ratio test *p* = 0.011), this finding did not remain statistically significant after accounting for the eight interaction tests performed across outcomes (Bonferroni‐adjusted *p* = 0.090). Therefore, the result should be considered exploratory and interpreted cautiously. No additional interactions were observed for any other outcome across exposure groups. Figure 1 illustrates change in all metabolic markers by PCOS‐medication group. Overall, mean change values were significantly different from zero (*p* < 0.05) for most marker‐group combinations, with the exception of change in fasting insulin for the neither medication/no PCOS group; change in HOMA‐IR, HbA1c and fasting insulin in the neither medication/PCOS group; and fasting insulin and ALT in the GLP‐1 only/no PCOS group (Figure 1; Table S2).

**FIGURE 1:**
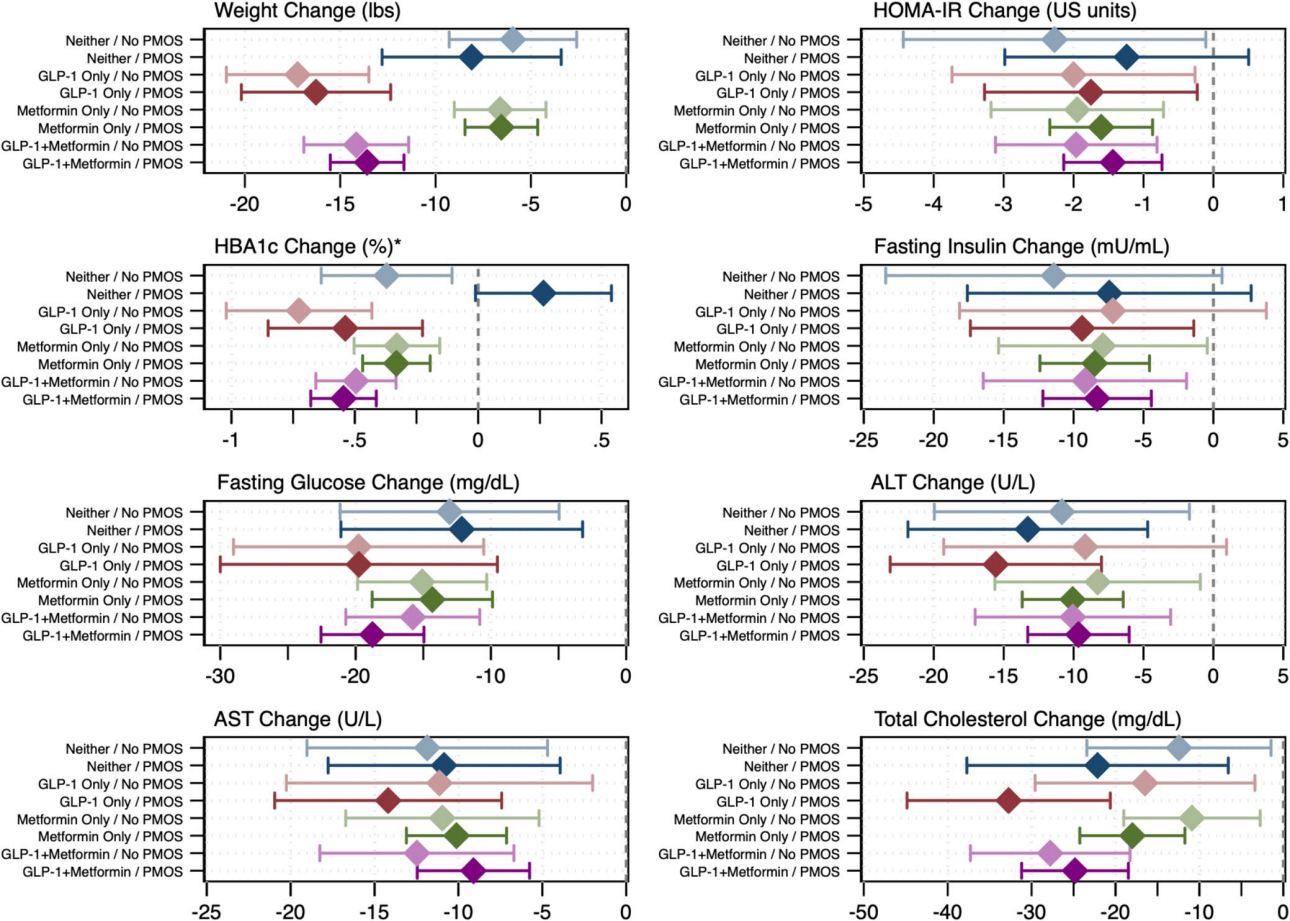
Adjusted mean outcome values (95% CI) by PMOS and medication group. Results adjusted for baseline value of the outcome, age, BMI, race/ethnicity, visit frequency and months of follow‐up. All mean change values were significantly different from zero (*p* < 0.05) except for changes in: Fasting Insulin for the Neither Medication/No PCOS group; changes in HOMA‐IR, HbA1c and Fasting Insulin in the Neither Medication/PCOS group; Fasting Insulin and ALT in the GLP‐1 Only/No PCOS group.

## 4 Discussion

This retrospective cohort analysis of nearly 1000 women initiating care through a specialized women's health telehealth platform demonstrates that clinically meaningful improvements across multiple metabolic markers are achievable in women with PCOS. We found clinical improvements that were equivalent in magnitude to those seen in women without PCOS, despite a significantly more adverse metabolic phenotype at baseline. To our knowledge, this represents one of the first real‐world analyses to characterize multi‐marker metabolic outcomes in women with PMOS within a multidisciplinary telehealth care model, addressing an important evidence gap [1].

The baseline differences between groups in our cohort are consistent with the known metabolic phenotype of PMOS and deserve consideration in interpreting the equivalence finding. Although patients with PMOS presented with only a slightly higher BMI, PMOS patients had substantially higher rates of insulin resistance, hyperinsulinemia and hepatic enzyme elevation—reflecting a pathophysiology wherein insulin resistance serves as a key driver of the disorder [8]. This pattern suggests that many PMOS patients in our cohort were at a more advanced stage of metabolic dysfunction, with elevated insulin and HOMA‐IR preceding frank glucose impairment. In the absence of intervention, this trajectory is not expected to stabilize: longitudinal studies demonstrate that hyperinsulinemia and insulin resistance worsen over time in women with PMOS, and intervention trial data show that the incidence of metabolic syndrome increases in care‐as‐usual comparator groups over 12 months of follow‐up [3, 4]. Further downstream, PMOS has been established as an important risk factor for cardiovascular disease, including stroke, myocardial infarction and composite cardiovascular disease outcomes [9]. Therefore, that women with this degree of metabolic complexity and heightened risk achieved substantial improvements equivalent to their non‐PMOS counterparts is a clinically important finding, suggesting a multidisciplinary model can be effective across a spectrum of metabolic risk and underlying diagnoses.

The magnitude of metabolic improvements observed is notable. Reductions of 14%–16% in insulin resistance markers, greater than 20% in liver enzymes and 6% in HbA1c over approximately 9 months of follow‐up compare favourably with outcomes reported in structured in‐person intervention programmes for PMOS patients [3]. Moreover, weight loss results with GLP‐1 RA's were broadly consistent with the real‐world weight loss outcomes in a large‐scale telehealth anti‐obesity medication programme [6]. The outcomes reported herein were achieved within a structured multidisciplinary model incorporating both physician and registered dietitian support, with participants attending a mean of 6.6 medical provider visits and 5.8 dietitian visits over the follow‐up period. Only about half of the participants were taking GLP‐1 RAs, with the remainder taking metformin alone or no medications. Importantly, improvements were observed even among participants receiving no pharmacological therapy: women with and without PMOS in the no‐medication group achieved reductions in weight, fasting glucose, liver enzymes and total cholesterol, suggesting that the structured multidisciplinary care model might contribute independently to metabolic improvement beyond the effects of medication alone. Given that medication access and tolerability vary considerably, our findings support the value of behaviour change and dietary optimization alongside or in lieu of pharmacological management.

The multi‐marker approach taken in the present analysis extends beyond weight‐centric reporting to capture a more comprehensive picture of metabolic health, which is particularly relevant for PMOS populations in whom insulin resistance and hepatic dysfunction may persist or predominate independently of weight change. The absence of a significant interaction between GLP‐1 RA use and PMOS status for weight loss suggests that the weight loss benefit of these medications generalizes to women with PMOS, a finding with practical implications given the high prevalence of overweight and obesity in this population. Notably, metformin alone was not associated with significant weight change in adjusted models, though the metformin alone group demonstrated significant HbA1c reduction, consistent with its established mechanism of action. A significant interaction between PMOS status and medication group was observed specifically for HbA1c, where PMOS patients without medication saw an increase in HbA1c, while non‐PMOS counterparts saw a decrease. This result may signal the metabolic challenges faced by those with PMOS, although given the number of interaction terms tested across eight outcomes, this finding should be interpreted cautiously as potentially reflecting Type I error.

Several limitations of this analysis warrant consideration. With a retrospective observational design, causation cannot be assigned and unmeasured confounding cannot be excluded. Medication group assignment was not randomized, and differences in clinical indication, severity of metabolic dysfunction, and patient preferences and goals are likely to have influenced prescribing patterns. Due to the study design, we are also unable to compare the impact from the multidisciplinary approach relative to working with a medical provider or dietitian alone. Misclassification bias may have also impacted our findings. PMOS status was inferred from ICD‐10 codes, assigned by providers instructed to use Rotterdam criteria for PMOS diagnosis. Nevertheless, it is possible that misclassification in both directions (incorrect diagnosis and missed diagnosis) could be present, which might have obscured finding a difference between groups. Similarly, pharmacologic treatment categories were based on EHR prescription data, which is likely subject to some degree of inaccuracy as well; it is likely that some patients without a prescription in our records obtained medication from alternate pathways, while some patients with a prescription may have ultimately chosen not to take the medication. Misclassification of treatment group would also bias our medication results toward the null. Although all patient groups in this study saw important metabolic improvements, the absence of a control group limits causal inference. Nevertheless, considered within the context of existing longitudinal data showing progressive worsening in untreated cohorts, the magnitude of improvements observed support a treatment effect. The study cohort was drawn from a single specialized telehealth platform, with a focus on PMOS and metabolic health, serving a predominantly privately insured population. Thus, our findings may not be easily extrapolated to under‐insured populations. Laboratory follow‐up was not available for all participants, and the requirement for at least one abnormal baseline laboratory value for inclusion in subgroup analyses, while methodologically appropriate, means that women with entirely normal baseline labs were excluded from those analyses. Patients were required to have at least 6 months of follow‐up data, meaning patients who were lost to follow‐up early in their treatment are not accounted for. We do not have reliable information regarding why patients may not have followed up after 6 months, though the excluded cohort had an overall less severe phenotype. Ultimately, our findings are only representative of patients who had engaged with the platform and pursued follow‐up testing. Notably, our follow‐up time of approximately 9 months was sufficient to capture meaningful metabolic change, but does not address longer‐term durability of outcomes, which remains an important question for both telehealth and PMOS‐specific care models.

These findings have meaningful implications for the design and delivery of metabolic care for women with PMOS. Recent guidance emphasizes the importance of integrated, multidisciplinary care addressing both metabolic and reproductive dimensions of the condition [1], yet access to such care remains a persistent barrier for many women [10]. The present data suggest that a structured telehealth platform incorporating medical provider oversight and dietitian support can achieve metabolic outcomes in women with PMOS that are clinically comparable to those in women without the condition—and comparable to what has been reported in more resource‐intensive in‐person programmes. Although we saw substantial weight loss for patients utilizing pharmacologic therapies, we also demonstrate that metabolic improvement is achievable without pharmacotherapy, albeit at a smaller scale. As telehealth platforms continue to scale and diversify their service offerings, real‐world evidence of this kind helps establish the evidence base for virtual metabolic care and supports policy and payer decisions that ultimately determine patient access.

## Author Contributions

**Heather G. Huddleston:** conceptualization, writing – original draft, methodology. **Rachel Blank:** conceptualization, writing – review and editing, resources. **Nandita Sommers:** conceptualization, writing – review and editing. **Nicole Hellman:** conceptualization. **Felice Ramallo:** conceptualization, data curation. **Tiffany Pham:** conceptualization, data curation. **Karla Lindquist:** methodology, formal analysis, writing – review and editing.

## Funding

Allara Health provided access to de‐identified clinical data. Analysis and interpretation were conducted independently by the authors.

## Disclosure

H.G.H. is Chief Medical Advisor at Allara Health, receives research funding, and holds stock options in the company, in addition to her faculty appointment at UCSF. R.B., N.S., N.H., F.R. and T.P. are employees of Allara Health. K.L. received a consulting fee from Allara Health for statistical analysis.

## Conflicts of Interest

The authors' competing interests are noted in the Disclosure section above.

## Supporting information


**Table S1:** Comparison of included versus excluded participants.
**Table S2:** Predicted mean changes (95% CI) by PMOS and medication status.

## Data Availability

The data that support the findings of this study are available from the corresponding author upon reasonable request.
