# Supplementary material for: Real‐World Metabolic Improvements in Women With and Without Polyendocrine Metabolic Syndrome via a Multidisciplinary Telehealth Care Platform
Source: Endocrinol Diabetes Metab. 2026 Oct 14;9(6):e70332. doi: 10.1002/edm2.70332 (PMC13662954; doi:10.1002/edm2.70332)
Supplement: Supplementary file 1 [file EDM2-9-e70332-s001.docx]

*Supplementary Table S1: comparison of included versus excluded participants*

| **Characteristic** | **All  (N=3117)** | **Included  (N=919)** | **Excluded  (N=2198)** | **P-value*** |
| --- | --- | --- | --- | --- |
| PCOS, n (%) | 1540 (49.4) | 557 (60.6) | 983 (44.7) | <0.001 |
| Age (years), mean (SD) | 34.7 (5.6) | 34.6 (5.6) | 34.7 (5.7) | 0.709 |
| Number of MD visits, mean (SD) | 4.3 (2.4) | 6.6 (2.3) | 3.3 (1.6) | <0.001 |
| Number of RD visits, mean (SD) | 3.6 (3.2) | 5.8 (3.6) | 2.7 (2.5) | <0.001 |
| Race, n (%): |  |  |  | 0.002 |
| Asian | 163 (5.2) | 50 (5.4) | 113 (5.1) |  |
| Black | 163 (5.2) | 49 (5.3) | 114 (5.2) |  |
| Hispanic | 317 (10.2) | 110 (12.0) | 207 (9.4) |  |
| White | 865 (39.4) | 393 (42.8) | 1258 (40.4) |  |
| Other/Multiethnic | 115 (3.7) | 41 (4.5) | 74 (3.4) |  |
| Unknown | 1101 (35.3) | 276 (30.0) | 825 (37.5) |  |
| BMI, mean (SD) | 33.6 (6.9) | 34.6 (7.2) | 33.2 (6.7) | <0.001 |
| **Outcome subgroup** |  |  |  |  |
| HOMA-IR > 4, n (%) | 1086 (34.8) | 401 (43.6) | 674 (30.7) | <0.001 |
| HbA1c > 5.7, n (%) | 577 (18.5) | 202 (22.0) | 371 (16.9) | <0.001 |
| Fasting insulin > 24, n (%) | 617 (19.8) | 218 (23.7) | 394 (17.9) | <0.001 |
| Fasting glucose > 100, n (%) | 469 (15.1) | 165 (18.0) | 303 (13.8) | <0.001 |
| ALT > 24, n (%) | 829 (26.6) | 270 (29.4) | 538 (24.5) | <0.001 |
| AST > 24, n (%) | 483 (15.5) | 140 (15.2) | 340 (15.5) | 0.949 |
| Total cholesterol > 200, n (%) | 1433 (46.0) | 403 (43.9) | 995 (45.3) | 0.222 |

*P-value by t-test for continuous variables and c2 for categorical variables

#### Supplementary Table S2: Predicted mean changes (95% CI) by PCOS and medication status

| **Medication / PCOS** | **Weight Change (lbs)** | **HOMA-IR Change (US units)** |
| --- | --- | --- |
| Neither / No PCOS | -5.9 (-9.3, -2.6) | -2.3 (-4.4, -0.1) |
| Neither / PCOS | -8.1 (-12.8, -3.4) | -1.2 (-3.0, 0.5) |
| GLP-1 Only / No PCOS | -17.2 (-21.0, -13.5) | -2.0 (-3.7, -0.3) |
| GLP-1 Only / PCOS | -16.3 (-20.2, -12.3) | -1.8 (-3.3, -0.2) |
| Metformin Only / No PCOS | -6.6 (-9.0, -4.2) | -1.9 (-3.2, -0.7) |
| Metformin Only / PCOS | -6.6 (-8.5, -4.6) | -1.6 (-2.3, -0.9) |
| GLP-1+Metformin / No PCOS | -14.2 (-16.9, -11.4) | -2.0 (-3.1, -0.8) |
| GLP-1+Metformin / PCOS | -13.6 (-15.5, -11.7) | -1.4 (-2.1, -0.7) |
| **Medication / PCOS** | **HbA1c Change (%)** | **Fasting Insulin Change (mU/mL)** |
| Neither / No PCOS | -0.4 (-0.6, -0.1) | -11.4 (-23.5, 0.7) |
| Neither / PCOS | 0.3 (0.0, 0.5) | -7.4 (-17.7, 2.8) |
| GLP-1 Only / No PCOS | -0.7 (-1.0, -0.4) | -7.2 (-18.2, 3.8) |
| GLP-1 Only / PCOS | -0.5 (-0.9, -0.2) | -9.4 (-17.4, -1.4) |
| Metformin Only / No PCOS | -0.3 (-0.5, -0.2) | -7.9 (-15.4, -0.4) |
| Metformin Only / PCOS | -0.3 (-0.5, -0.2) | -8.5 (-12.4, -4.5) |
| GLP-1+Metformin / No PCOS | -0.5 (-0.7, -0.3) | -9.2 (-16.5, -1.9) |
| GLP-1+Metformin / PCOS | -0.5 (-0.7, -0.4) | -8.3 (-12.2, -4.4) |
| **Medication / PCOS** | **Fasting Glucose Change (mg/dL)** | **ALT Change (U/L)** |
| Neither / No PCOS | -13.1 (-21.2, -4.9) | -10.8 (-20, -1.7) |
| Neither / PCOS | -12.2 (-21.2, -3.2) | -13.3 (-21.9, -4.7) |
| GLP-1 Only / No PCOS | -19.8 (-29.1, -10.5) | -9.2 (-19.3, 1.0) |
| GLP-1 Only / PCOS | -19.8 (-30.1, -9.4) | -15.6 (-23.1, -8) |
| Metformin Only / No PCOS | -15.1 (-19.9, -10.3) | -8.3 (-15.7, -0.9) |
| Metformin Only / PCOS | -14.3 (-18.8, -9.9) | -10.1 (-13.7, -6.4) |
| GLP-1+Metformin / No PCOS | -15.8 (-20.8, -10.8) | -10.0 (-17.1, -3.0) |
| GLP-1+Metformin / PCOS | -18.8 (-22.6, -14.9) | -9.6 (-13.3, -6.0) |
| **Medication / PCOS** | **AST Change (U/L)** | **Total Cholesterol Change (mg/dL)** |
| Neither / No PCOS | -11.9 (-19.1, -4.6) | -12.4 (-23.5, -1.4) |
| Neither / PCOS | -10.9 (-17.8, -3.9) | -22.1 (-37.8, -6.5) |
| GLP-1 Only / No PCOS | -11.1 (-20.3, -1.9) | -16.5 (-29.6, -3.3) |
| GLP-1 Only / PCOS | -14.2 (-21, -7.4) | -32.7 (-44.9, -20.6) |
| Metformin Only / No PCOS | -11.0 (-16.8, -5.1) | -10.9 (-19.0, -2.7) |
| Metformin Only / PCOS | -10.1 (-13.1, -7.1) | -18.0 (-24.3, -11.7) |
| GLP-1+Metformin / No PCOS | -12.5 (-18.3, -6.6) | -27.8 (-37.3, -18.2) |
| GLP-1+Metformin / PCOS | -9.1 (-12.5, -5.7) | -24.8 (-31.2, -18.4) |

Results adjusted for baseline value of the outcome, age, BMI, race/ethnicity, visit frequency, and months of follow-up

#### All mean change values were significantly different from zero (p<0.05) except for changes in: Fasting Insulin for the Neither Medication/No PCOS group; changes in HOMA-IR, HbA1c, and Fasting Insulin in the Neither Medication/PCOS group; Fasting Insulin and ALT in the GLP-1 Only/No PCOS group.
